# 923. REACH for Resilience Outcomes-A Map of Success; Short Term Analysis of Post Burn Survivor Retreats

**DOI:** 10.1093/jbcr/irag033.448

**Published:** 2026-04-06

**Authors:** Roselle E Crombie, Daniel W Chacon, Rebekah R Allely, Cindy Rutter

**Affiliations:** Connecticut Burn Center/Yale New Haven Health, Westport, CT; Phoenix Society for Burn Survivors, Fresno, CA; Medstar Washington Hospital Center, Reston, VA; 1 Amazing Life, Carlsbad, CA

## Abstract

**Introduction:**

Community-based and recreational programming is widely used in burn survivorship with short-term evaluations often showing gains in coping, connection, and well-being. Yet the durability of these benefits and the translation of skills into everyday behavior remain underreported. To address this gap, we pursued two aims: (1) to quantify resilience 6–12 months post-retreat, operationalized as resilience beliefs (10-item scale) and the prevalence of resilience behaviors (Behavioral Resilience Indicators, BRI); and (2) to synthesize participant-reported meaningful changes and ongoing support needs.

**Methods:**

attendees from two adult retreats (n = 20) were invited to complete a follow-up survey 6–12 months after participation; Fifteen responded (75%). The 10-item resilience scale used a 5-point Likert response set (not true at all, rarely, sometimes, often, nearly always). The BRI comprised 10 yes/no indicators of resilience behaviors practiced in the prior six months. Analyses were descriptive. Three open-ended questions were coded thematically. Baseline data were not available for these cohorts and will be collected prospectively.

**Results:**

Sample (n = 15). Age 25–61 years (mean 40.0, SD 11.18).

Resilience beliefs (10-item scale, categorical). All 10/10 items reached ≥73% High endorsement (range 73%–100%). The lowest-endorsed items were Q8 and Q10 (11/15 High, 73% each). The highest-endorsed items were Q1 (adapting to change) and Q6 (achieving goals despite obstacles), with 15/15 High (100%). Responses outside the High category were predominantly “sometimes” rather than **“rarely/not at all.”

Behavioral resilience (BRI). All 10/10 behaviors were endorsed by ≥80% of respondents (mean ≈88%, range 80–100%). Examples: reached out to a supportive person 86.7%; practiced gratitude 86.7%; used positive self-talk 93.3%; reframed setbacks 93.3%; breathing/grounding 86.7%; accomplished a personal/professional goal 100%.

Belief–behavior alignment. High endorsement of Q6 (100%) aligned with 100% endorsement of the BRI item “accomplished a personal/professional goal,” indicating coherence between perceived capacity and enacted behavior at follow-up.

**Conclusions:**

At 6–12 months post-retreat, survivors reported broadly high resilience beliefs (≥73% high across all items) and frequent use of resilience behaviors (≈88% across indicators), including universal goal attainment in the follow-up window. These follow up only data suggest durable skill use and feasibility of longitudinal contact. Future cohorts will incorporate standardized baseline measures and brief booster touchpoints, with particular attention to emotion regulation (the relatively lower-endorsed belief items).

**Applicability of Research to Practice:**

Tracking in a standard method of post intervention data is essential to understanding it's role in survivor recovery.

**Funding for the study:**

N/A.

